# An Outer Membrane Vesicle-Based Vaccine Combined with alb-Flt3L Promotes Durable Antitumor Immunity in HPV-Associated Cancer

**DOI:** 10.3390/vaccines14080707

**Published:** 2026-08-18

**Authors:** Yining Liu, Yichu Xu, Yu-Cheng Chang, Ya-Chea Tsai, Tzyy-Choou Wu, Chien-Fu Hung

**Affiliations:** 1 Department of Pathology, Johns Hopkins University School of Medicine, Baltimore, MD 21287, USA; yliu661@jh.edu (Y.L.); yichuxu2@gmail.com (Y.X.); 108004@w.tmu.edu.tw (Y.-C.C.); ytsai5@jhmi.edu (Y.-C.T.); 2 Department of Biochemistry and Molecular Biology, Johns Hopkins University Bloomberg School of Public Health, Baltimore, MD 21205, USA; 3 Department of Oncology, Johns Hopkins University School of Medicine, Baltimore, MD 21287, USA; 4 Department of Obstetrics and Gynecology, Johns Hopkins University School of Medicine, Baltimore, MD 21287, USA; 5 Department of Molecular Microbiology and Immunology, Johns Hopkins University School of Medicine, Baltimore, MD 21287, USA

**Keywords:** HPV-associated cancer, cancer vaccine, outer membrane vesicle, Flt3L, antitumor immunity

## Abstract

**Background/Objectives**: Human papillomavirus (HPV)-associated cancers remain a major global health burden, and no therapeutic cancer vaccine has yet been approved. Oncoprotein E7 plays a key role in tumor initiation and progression and has been identified as a potential target. Here, we aim to improve the efficacy and durability of an outer membrane vesicle (OMV)-based E7-targeted vaccine, SOMV-9RE7, through alb-Flt3L combination therapy. **Methods**: The antitumor efficacy and durability of the combination therapy were evaluated in low-burden and high-burden HPV-positive TC-1 tumor-bearing mouse models. Systemic and local immune responses were investigated by flow cytometry. **Results**: Combination with alb-Flt3L improved the tumor control and prolonged the therapeutic durability of SOMV-9RE7 compared with monotherapy groups, with more than half of the treated mice surviving beyond 60 days. This combination strategy enhanced E7-specific CD8^+^ T cell immunity in peripheral blood and the spleen, reduced myeloid-derived suppressor cell (MDSC)-mediated immunosuppression, promoted splenic T cell memory formation, and reshaped the tumor microenvironment. **Conclusions**: Combining vaccine SOMV-9RE7 with alb-Flt3L improves antitumor efficacy and durability. This therapeutic benefit is associated with both systemic and local immune remodeling, supporting the combination therapy as a promising strategy for therapeutic cancer vaccines.

## 1. Introduction

Human papillomaviruses (HPVs) are double-stranded DNA viruses that infect human epithelial cells [1]. Persistent high-risk HPV infection is associated with various malignancies, including cervical, anal, oropharyngeal, and other anogenital cancers [2,3]. These HPV-associated cancers account for around 5% of cancer cases worldwide each year, remaining a substantial global health burden [4].

The oncogenic potential of high-risk HPV strains is largely driven by the integration of viral oncogenes, resulting in the continuous expression of oncoproteins [5]. Among these, the E7 oncoprotein plays a crucial role in tumor progression by promoting cell cycle dysregulation and immune evasion [6,7,8]. Based on its persistent expression and function, E7 has been recognized as a potential therapeutic target for HPV-associated cancers.

Conventional cancer treatments, including surgery, chemotherapy, and radiotherapy, are often limited by poor specificity, toxicity, therapeutic resistance, and recurrence [9]. Immunotherapy targeting tumor-specific neoantigens or tumor-associated antigens, such as E7, provides an alternative strategy. Various vaccine platforms have been explored for HPV and HPV-associated cancers [10]. While some of these vaccines have shown partial success, no therapeutic vaccine has yet been approved for the treatment of established HPV-associated cancers [11,12].

Bacteria-driven platforms have recently emerged as potential strategies due to their intrinsic immunostimulatory properties and delivery capacity [13,14,15]. Outer membrane vesicles (OMVs) are non-replicable nanoscale vesicles derived from Gram-negative bacteria [16]. OMVs carry various pathogen-associated molecular patterns (PAMPs), which can activate host innate immune response and promote antitumor immunity [17,18,19]. In previous work, we developed an HPV-associated E7-displaying Salmonella-derived OMV vaccine, termed SOMV-9RE7. In this formulation, a nine-arginine motif was fused to the E7 peptide as a cationic anchoring sequence to enhance peptide association and antigen display [20]. This vaccine has been shown to stimulate antigen-specific antitumor immune responses in the TC-1 tumor model [21]. However, the durability of SOMV-9RE7 as a monotherapy remains limited. Although SOMV-9RE7 monotherapy produced strong initial tumor control, its antitumor effect was not sustained long term. This indicates that efficient antigen delivery alone may not be sufficient for optimal efficacy.

Supporting dendritic cells (DCs) is a potential strategy for enhancing antitumor efficacy. DCs play a crucial role in capturing, processing, and presenting tumor-associated antigens to initiate T cell antitumor immunity [22]. Fms-like tyrosine kinase 3 ligand (Flt3L) is a key growth factor that promotes DC expansion and homeostasis [23,24,25], and albumin fusion has been shown to prolong its in vivo persistence [26]. Therefore, in this study, we investigated whether the combination therapy of SOMV-9RE7 and alb-Flt3L could enhance therapeutic efficacy and durability. We also further characterized the systemic and local immune alteration induced by this combination strategy.

## 2. Materials and Methods

### 2.1. Cell Preparation

E7-expressing TC-1 tumor cells were grown in vitro in RPMI 1640 media containing 10% (*v*/*v*) fetal bovine serum, 1 mM sodium pyruvate, 2 mM L-glutamine, 2 mM non-essential amino acids, 50 units/mL of penicillin/streptomycin, and 0.1% 2-mercaptoethanol under 37 °C with 5% CO_2_.

### 2.2. Peptide Synthesis

The vaccine peptide used in this study, designated as 9RE7, consists of the HPV-16 E749-57 epitope fused to a poly-arginine anchoring motif (RRRRRRRRR-RAHYNIVTF). The binding properties and immunogenicity of 9RE7 were previously characterized [21]. Synthesis was performed at a purity of over 90% (GenScript, Piscataway, NJ, USA).

### 2.3. Bacteria-Derived Outer Membrane Vesicles

*Salmonella* SL7207 was grown overnight in LB broth at 37 °C. The next day, 1 mL of the overnight culture was added to 9 mL of fresh LB broth and incubated until the OD600 reached 0.5. The freshly cultured Salmonella was inoculated into 250 mL of fresh LB medium at 37 °C for 16 h. The supernatant was collected after centrifugation and filtered through a 0.45 μm Membrane Filter (MilliporeSigma, St. Louis, MO, USA). The filtered medium was transferred to ultracentrifuge tubes and centrifuged at 150,000 RPM for 3 h. The particles at the bottom of the tubes were collected, resuspended in 1 mL PBS, and stored at −80 °C. OMV yield was calculated using the Bio-Rad protein assay (Bio-Rad Laboratories, Hercules, CA, USA) with a concentration of approximately 1.5 mg/mL.

### 2.4. SOMV-9RE7 Generation

SOMV-9RE7 was prepared by mixing SOMV and 9RE7 peptide in PBS buffer, followed by vortexing for 30 min. Unbound peptides were removed using a 50kD Amicron Ultra Centrifugal Filter (MilliporeSigma, St. Louis, MO, USA).

### 2.5. alb-Flt3L Generation and Expression

Flt3L was first amplified by PCR from a cDNA template plasmid synthesized from Genscript (Genscript, Piscataway, NJ, USA). The amplified product was then cloned into the EcoRI/BamHI sites of pcDNA3-alb. The construction was confirmed by DNA sequencing. The alb-Flt3L protein was expressed in Expi293F cells using the Expi293F expression system kit (Thermo Fisher Scientific, Waltham, MA, USA) and was purified using a HiTrap Albumin column (GE Healthcare Life Sciences, Chicago, IL, USA).

### 2.6. Mice

Female C57BL/6 mice (6–8 weeks old) were purchased from Charles Rivers Laboratories and maintained under specific pathogen-free conditions at the Johns Hopkins University School of Medicine Animal Facility. All animal procedures were performed in accordance with protocols approved by the Johns Hopkins Institutional Animal Care and Use Committee (IACUC; protocol MO23M84, approved on 20 March 2023). All experiments were conducted in compliance with institutional and national guidelines for the care and use of laboratory animals.

### 2.7. TC-1 Tumor-Bearing Mouse Model and Treatment

A total of 5 × 10^4^ or 1 × 10^5^ TC-1 cells in 100 μL PBS were injected subcutaneously into 6–8-week-old female C57BL/6 mice in the lower right abdomen to establish low- or high-burden HPV-associated tumor models. Tumor volumes were measured and calculated three times per week. At the indicated time points, alb-Flt3L was administered intraperitoneally at a dose of 50 μg per mouse. Tumor-bearing mice were vaccinated subcutaneously on the contralateral side, away from the tumor site, with SOMV-9RE7 (10 μg of 9RE7 peptides and 10 μg of SOMV).

### 2.8. Flow Cytometry Analyses

Peripheral blood samples were collected from mice at the indicated time points. PBMCs were isolated following red blood cell lysis. For tumor sample preparation, tumor tissues were digested with Collagenase I, Collagenase IV, and DNase I; mechanically dissociated using a gentleMACS Dissociator (Miltenyi Biotec, Gaithersburg, MD, USA); and incubated for 20 min. After centrifugation and buffer exchange, tumor-infiltrating leukocytes were enriched by Ficoll-Paque Plus (GE Healthcare Life Sciences, Chicago, IL, USA) density gradient centrifugation. Cells at the interface were collected. For spleen sample preparation, spleens were dissociated through a Corning 70 μm cell strainer and suspended in RPMI medium. Red blood cells were lysed, and splenocytes were collected. After counting, cells were plated at appropriate numbers and stained with antibodies against CD45, CD3, CD8α, CD11b, Ly6G, Ly6C, CD44, CD62L, and E7 tetramer, as appropriate for each flow cytometry panel. Detailed gating strategies and the antibody-fluorophore combinations are provided in Supplementary Figures S1–S3. Data were analyzed using FlowJo v10.8 software (BD Life Sciences, Franklin Lakes, NJ, USA).

### 2.9. Statistical Analysis

All data are expressed as the mean ± standard error of the mean (SEM). Kaplan–Meier survival curves were generated to assess survival, and the log-rank test was performed for survival analysis. Data were analyzed by a one-way ANOVA with Šídák’s post hoc multiple comparisons test and a two-way ANOVA with Tukey’s post hoc multiple comparisons test using GraphPad Prism 10 software (GraphPad, San Diego, CA, USA). All *p* ≤ 0.05 were considered significant (* *p* < 0.05; ** *p* < 0.01; *** *p* < 0.001; **** *p* < 0.0001; ns, not significant).

## 3. Results

### 3.1. alb-Flt3L Enhances the Antitumor Efficacy and Durability of SOMV-9RE7 in a Low-Burden Mouse Tumor Model

**Figure 1:**
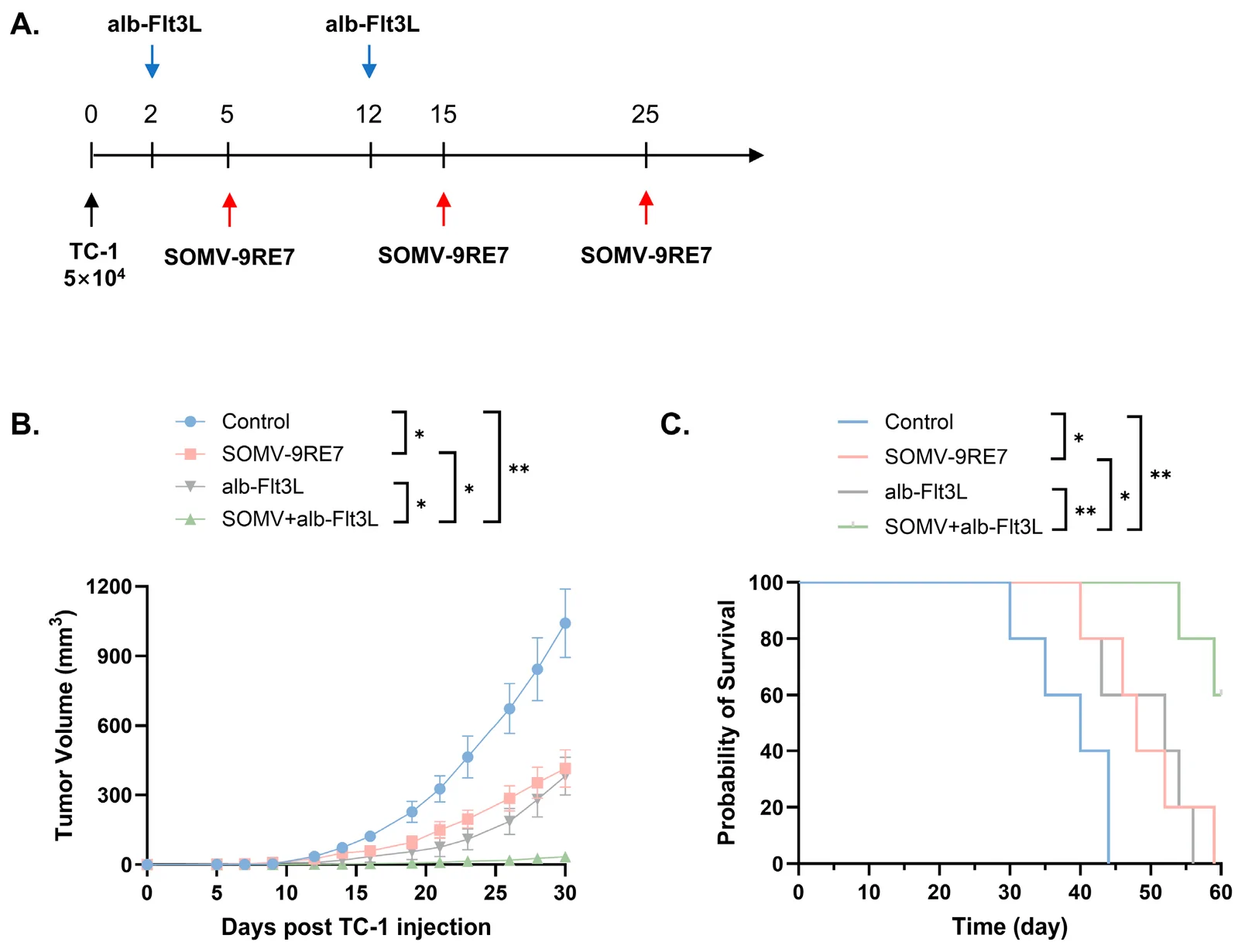
Identification of alb-Flt3L as effective partner for SOMV-9RE7 combination therapy. (**A**) Experimental design schematic. Mice (*n* = 5) were subcutaneously inoculated with 5 × 10^4^ TC-1 tumor cells. SOMV-9RE7 and alb-Flt3L were administered according to indicated schedule. (**B**) Tumor growth curve of untreated mice and mice treated with SOMV-9RE7 monotherapy, alb-Flt3L monotherapy, or combination of SOMV-9RE7 and alb-Flt3L (SOMV+alb-Flt3L). (**C**) Kaplan-Meier survival curve of four groups. Data were presented as mean ± SEM and were analyzed by two-way ANOVA with Tukey’s post hoc multiple comparisons test for tumor growth (**B**) and log-rank (Mantel-Cox) test for survival curve (**C**). * *p* < 0.05, ** *p* < 0.01.

To improve the efficacy and durability of SOMV-9RE7, we evaluated the combination of SOMV-9RE7 and alb-Flt3L in a low-burden HPV-positive TC-1 tumor model (Figure 1A) [26]. Tumor growth and survival were monitored. Compared with vaccine monotherapy, the combination of SOMV-9RE7 and alb-Flt3L significantly improved tumor control (Figure 1B). More importantly, the combination therapy significantly prolonged survival. More than half of the treated mice survived beyond 60 days, indicating improved therapeutic durability (Figure 1C).

### 3.2. SOMV-9RE7 Plus alb-Flt3L Maintains Antitumor Efficacy in a High-Burden Mouse Tumor Model

**Figure 2:**
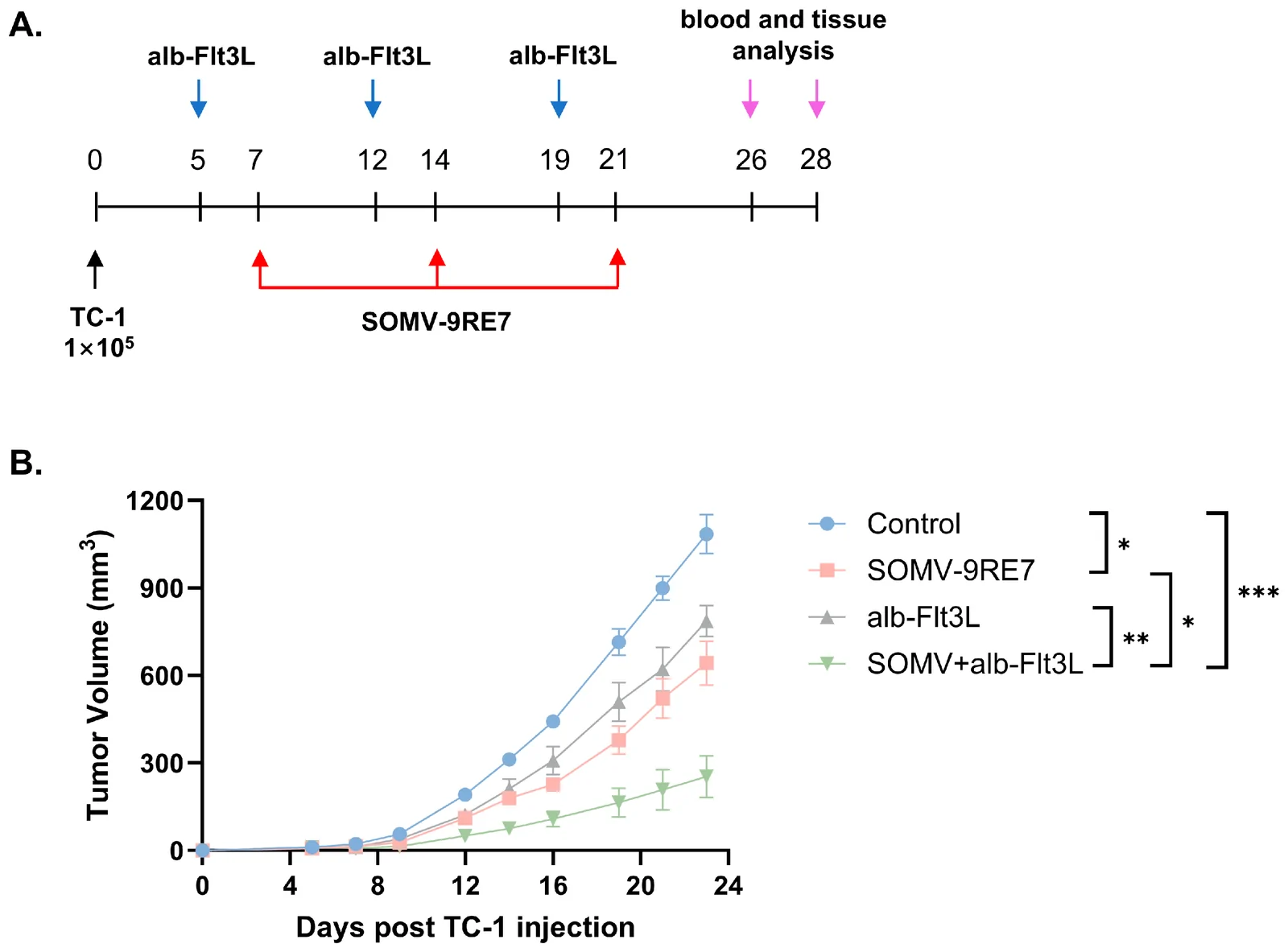
SOMV-9RE7 plus alb-Flt3L maintains antitumor efficacy in high-burden TC-1 tumor model. (**A**) Experimental design schematic. Mice (*n* = 5 per group) were inoculated subcutaneously with 1 × 10^5^ TC-1 tumor cells. SOMV-9RE7 and alb-Flt3L were administered according to indicated schedule. (**B**) Tumor growth curve of untreated mice and mice treated with SOMV-9RE7 monotherapy, alb-Flt3L monotherapy, or combination of SOMV-9RE7 and alb-Flt3L (SOMV+alb-Flt3L). Data were presented as mean ± SEM and were analyzed by two-way ANOVA with Tukey’s post hoc multiple comparisons test. * *p* < 0.05; ** *p* < 0.01; *** *p* < 0.001.

To further validate the therapeutic potential of this combination strategy, we next evaluated it in a high-burden TC-1 tumor model (Figure 2A). In this setting, the tumor inoculation dose was doubled to 1 × 10^5^, and the interval between tumor grafting and treatment was extended to increase the tumor challenge. Consistent with the results in the low-burden model, SOMV-9RE7 plus alb-Flt3L significantly improved tumor control even under these more challenging conditions compared with the control and monotherapy groups. Notably, this antitumor effect was sustained throughout the observation period (Figure 2B).

### 3.3. Combination Therapy Enhances Systemic Antigen-Specific CD8^+^ T Cell Responses and Attenuates MDSC-Mediated Immunosuppression

**Figure 3:**
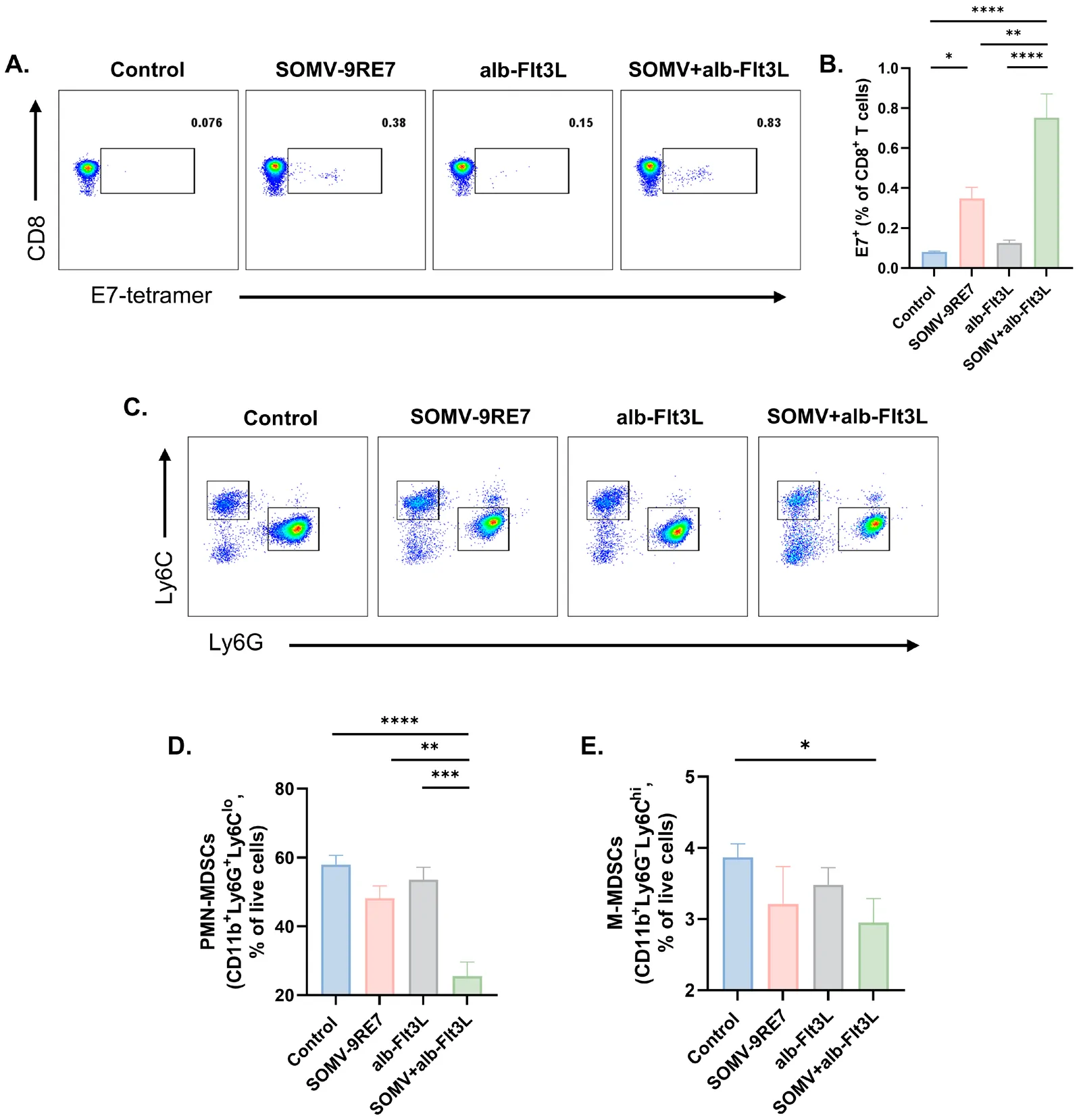
Combination therapy enhances systemic E7-specific CD8^+^ T cell responses and reduces MDSCs in peripheral blood. Blood samples were collected on day 26 after tumor inoculation and analyzed by flow cytometry (*n* = 5 per group). (**A**,**B**) Representative flow cytometry plots (**A**) and bar graph (**B**) showing frequency of E7-specific CD8^+^ T cells in CD8^+^ T cells. Peripheral blood mononuclear cells (PBMCs) were stained with APC-A750-conjugated CD8α antibody and PE-conjugated HPV16 E7 tetramer. (**C**–**E**) Representative flow cytometry plots (**C**) and bar graphs (**D**,**E**) showing MDSC population and subset identification. PMN-MDSCs (**D**) were defined as CD11b^+^ Ly6G^+^ Ly6C^lo^ cells, and M-MDSCs (**E**) were defined as CD11b^+^ Ly6G^−^ Ly6C^hi^ cells. Data are presented as mean ± SEM and were analyzed by one-way ANOVA with Šídák’s post hoc multiple comparisons test (**B**,**D**) or Student’s *t* test (**E**). * *p* < 0.05, ** *p* < 0.01, *** *p* < 0.001, **** *p* < 0.0001.

To evaluate the systemic immune responses induced by the combination therapy, peripheral blood was collected on day 26 after tumor inoculation and analyzed by flow cytometry. As shown in Figure 3A,B, the vaccine monotherapy significantly increased the frequency of E7-specific CD8^+^ T cells compared with the control, while this antigen-specific immune response was further enhanced by the addition of alb-Flt3L. The mice treated with the combination therapy showed the highest frequency of E7-specific cytotoxic T lymphocytes (CTLs) among the four groups. Consistent with the improved tumor control, these results indicate that SOMV-9RE7 plus alb-Flt3L enhances antigen-specific antitumor immunity.

Overcoming immunosuppression induced by the tumor is also essential for maintaining durable antitumor efficacy. Therefore, we next characterized MDSCs, a major immunosuppressive population in tumor-bearing hosts, to determine whether this combination strategy altered systemic immunosuppression (Figure 3C). The combination therapy significantly reduced the PMN-MDSCs compared with the control and monotherapy groups (Figure 3D). In addition, a significant decrease in M-MDSCs was observed in the combination therapy group compared with untreated mice (Figure 3E). These findings indicate that the combination therapy effectively reduces MDSC-mediated systemic immunosuppression.

### 3.4. Combination Therapy Enhances Splenic T Cell Expansion and Antigen-Specific Cytotoxic Responses

**Figure 4:**
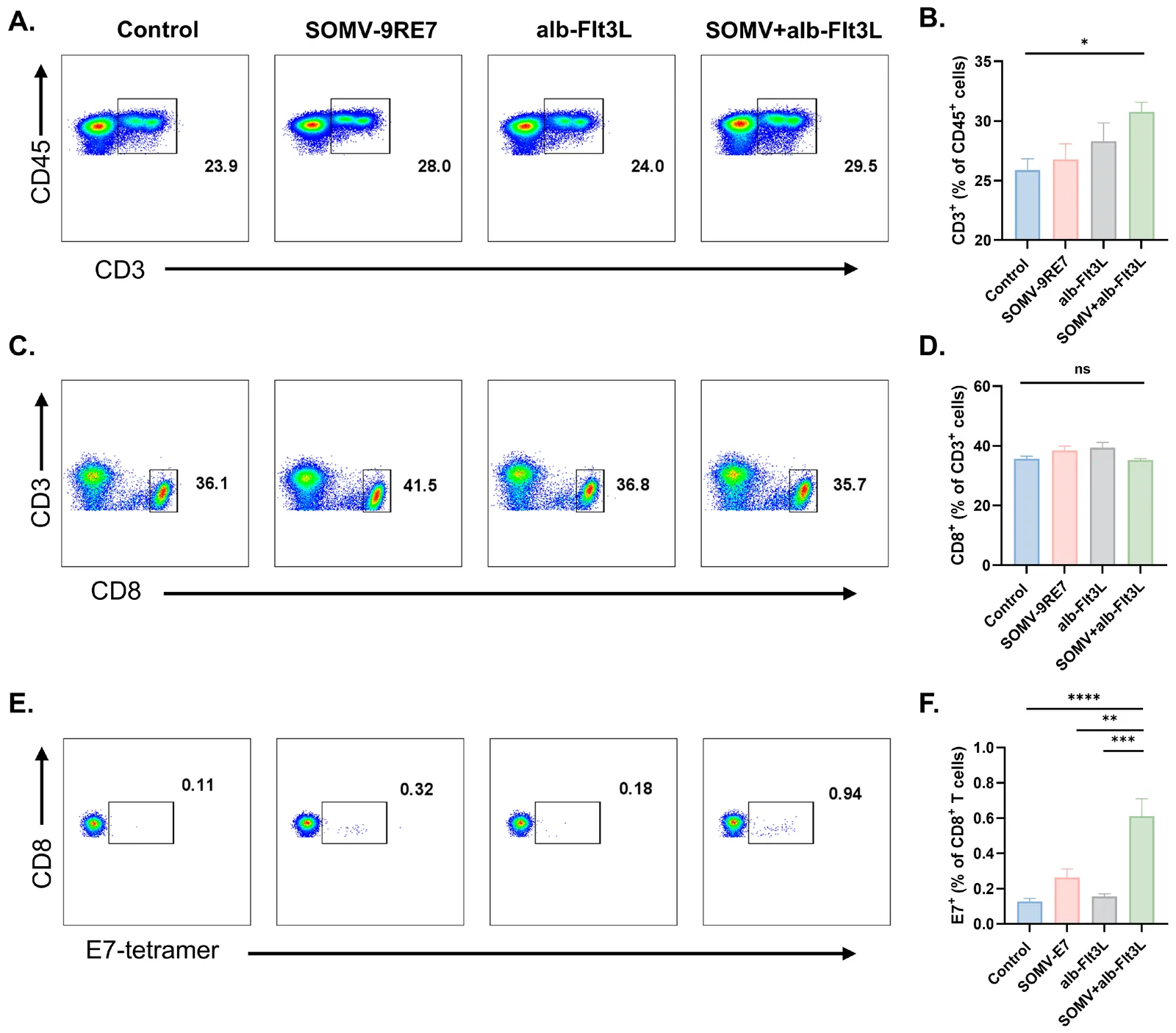
Combination therapy enhances splenic T cell expansion and antigen-specific T cell immune responses. Splenic cells were collected on day 28 after tumor inoculation and analyzed by flow cytometry (*n* = 5 per group). (**A**,**B**) Representative flow cytometry plots (**A**) and bar graph (**B**) of CD3^+^ T cells gated on CD45^+^ cells in spleens. (**C**,**D**) Representative flow cytometry plots (**C**) and bar graph (**D**) of CD8^+^ T cells gated on CD3+ cells. (**E**,**F**) Representative flow cytometry plots (**E**) and bar graph (**F**) of E7 tetramer^+^ T cells gated on CD8^+^ cells. Data are presented as mean ± SEM and were analyzed by one-way ANOVA with Šídák’s post hoc multiple comparisons test. * *p* < 0.05; ** *p* < 0.01; *** *p* < 0.001; **** *p* < 0.0001; ns, not significant.

To further examine the systemic adaptive immune response induced by the combination therapy, splenocytes were collected on day 28 after tumor inoculation and analyzed by flow cytometry. Compared with the control group, the combination therapy group showed a significant increase in CD45^+^ CD3^+^ T cells, whereas alb-Flt3L monotherapy showed a trend toward an increase (Figure 4A,B). The CD8^+^/CD3^+^ ratio remained consistent, with no significant difference observed among the four groups (Figure 4C,D). In addition, SOMV-9RE7 monotherapy showed an increasing trend in E7-specific CD8^+^ T cells, whereas the combination treatment significantly enhanced this population (Figure 4E,F). These results indicate that the combination of vaccine and alb-Flt3L enhances splenic T cell expansion and antigen-specific antitumor immune response.

### 3.5. Combination Therapy Promotes a Memory-Associated Phenotype in Splenic T Cells

**Figure 5:**
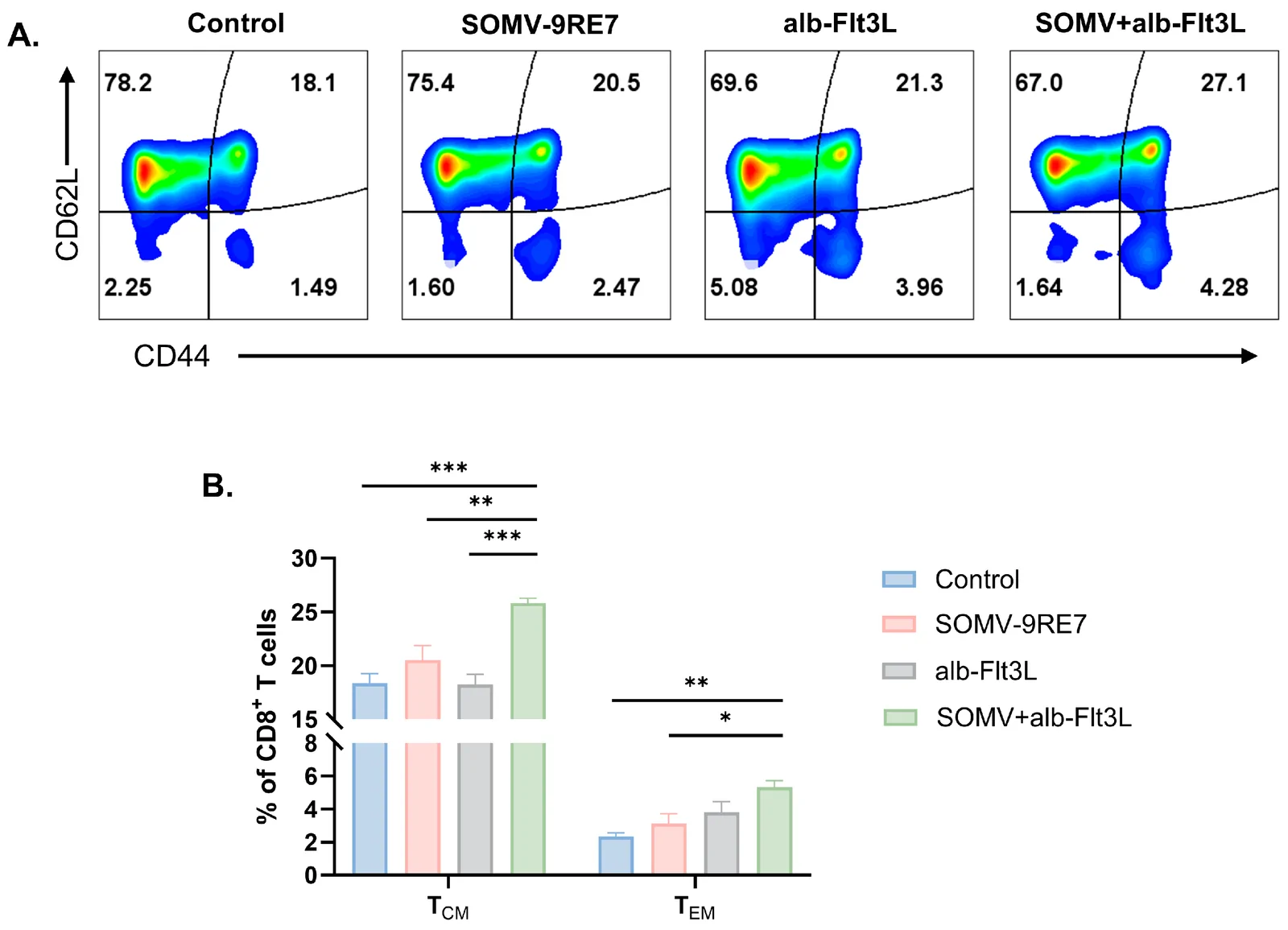
Combination therapy promotes T cell memory-associated phenotypes. Splenic cells were collected on day 28 after tumor inoculation and analyzed by flow cytometry (*n* = 5 per group). (**A**,**B**) Representative flow cytometry plots (**A**) and bar graph (**B**) of memory subsets of splenic CD8^+^ T cells. T_EM_ cells were defined as CD44^hi^ CD62L^lo^ cells. T_CM_ cells were defined as CD44^hi^ CD62L^hi^ cells. Data are presented as mean ± SEM and were analyzed by one-way ANOVA with Šídák’s post hoc multiple comparisons test. * *p* < 0.05, ** *p* < 0.01, *** *p* < 0.001.

To evaluate the long-term protective potential of this combination strategy, we next characterized the memory subsets of splenic T cells based on CD44 and CD62L expression [27]. Within splenic CD44^+^ CD8^+^ T cells, central memory T cells (T_CM_; CD44^hi^ CD62L^hi^) were the predominant population and were significantly increased in the combination group compared with the control and monotherapy groups. Although effector memory T cells (T_EM_; CD44^hi^ CD62L^lo^) were less abundant, combination therapy also significantly increased their frequency, with an approximately twofold increase compared with the control group (Figure 5A,B). These results suggest that the combination therapy promotes a memory-associated phenotype among splenic CD8^+^ T cells.

### 3.6. Combination Therapy Enhances Tumor Immune Cell Infiltration and Antigen-Specific CD8^+^ T Cell Responses

**Figure 6:**
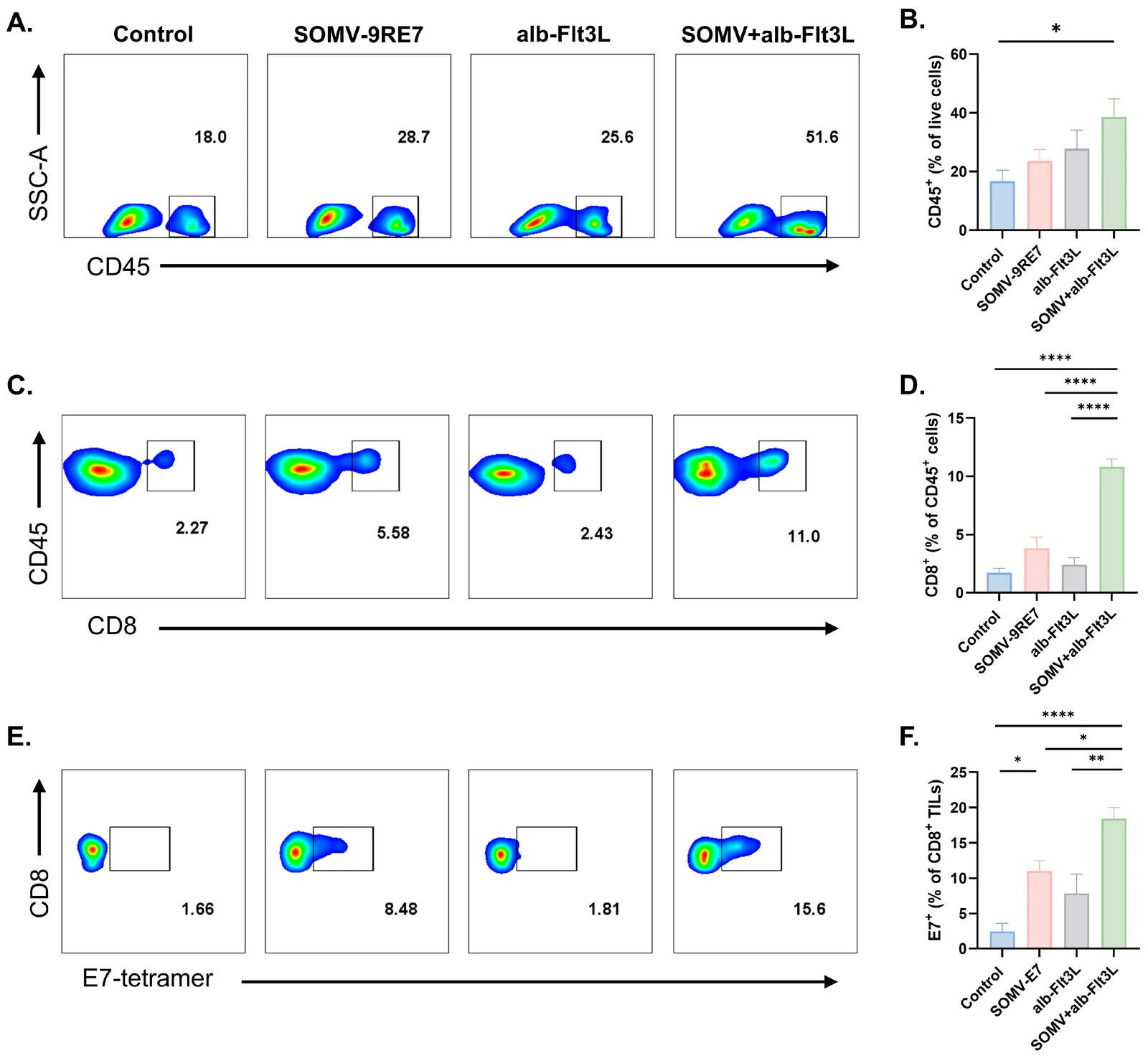
Combination therapy enhances immune cell infiltration and antigen-specific CD8^+^ T cell responses in TME. TILs were collected on day 28 after tumor inoculation and analyzed by flow cytometry (*n* = 5 per group). (**A**,**B**) Representative flow cytometry plots (**A**) and bar graph (**B**) of CD45^+^ TILs gated on live cells. (**C**,**D**) Representative flow cytometry plots (**C**) and bar graph (**D**) of CD8^+^ T cells gated on CD45^+^ cells. (**E**,**F**) Representative flow cytometry plots (**E**) and bar graph (**F**) of E7 tetramer^+^ T cells gated on CD8+ cells. Data are presented as mean ± SEM and were analyzed by one-way ANOVA with Šídák’s post hoc multiple comparisons test. * *p* < 0.05, ** *p* < 0.01, **** *p* < 0.0001.

Finally, we evaluated tumor-infiltrating lymphocytes (TILs) to determine whether combination therapy could enhance local antitumor immune responses within the tumor microenvironment (TME). Tumors were harvested on day 28 after tumor inoculation and processed into single mononuclear cells for FACS analysis. Compared with the control group, SOMV-9RE7 and alb-Flt3L monotherapy did not significantly alter the frequency of CD45^+^ TILs. In contrast, the combination therapy significantly increased the frequency of CD45^+^ TILs (Figure 6A,B). An approximately twofold expansion relative to the control group was observed, indicating enhanced immune cell infiltration within the TME. In addition, the proportion of CD8^+^ T cells among CD45^+^ infiltrating cells was also significantly higher in the combination group than in the control and monotherapy groups (Figure 6C,D).

Moreover, an analysis of antigen-specific immune response revealed that the SOMV-9RE7 monotherapy significantly increased the frequency of E7-specific CD8^+^ T cells compared with the control group, and this response was further enhanced by the combination therapy (Figure 6E,F). Together, these results reveal that the combination strategy most effectively enhances immune infiltration within the TME and promotes antigen-specific T cell antitumor immunity at the tumor site.

## 4. Discussion

In this study, we show that combining vaccine SOMV-9RE7 with alb-Flt3L improves antitumor efficacy and durability. This therapeutic benefit was observed in both low-burden and high-burden HPV-positive TC-1 tumor models and was associated with immune remodeling at the systemic and local levels. Specifically, the combination of SOMV-9RE7 and alb-Flt3L enhanced antigen-specific T cell antitumor immunity, reduced systemic immunosuppression, promoted T cell memory formation, and reshaped the TME. Together, these findings suggest that SOMV-9RE7 and alb-Flt3L function as a promising strategy against HPV-associated cancers.

The limited durability of SOMV-9RE7 monotherapy suggests that efficient antigen delivery alone may be insufficient for robust antitumor efficacy. Effective antigen recognition, presentation, and T cell priming are also required for optimal vaccination. As a growth factor for hematopoietic progenitor cells, Flt3L plays a key role in DC expansion and homeostasis, which makes it a rational partner and activator [23,24,25]. In addition, albumin fusion improves the in vivo persistence of alb-Flt3L, resulting in sustained immunomodulatory activity. The translational potential of Flt3L-based immunomodulation is further supported by clinical evidence. A Phase II clinical trial has shown that recombinant human Flt3L (CDX-301), in combination with cancer vaccination, expanded multiple dendritic cell subsets and enhanced vaccine-induced immune responses in patients with melanoma [28]. Together, these findings support the rationale for combining alb-Flt3L with SOMV-9RE7 and may help explain the improved efficacy and durability observed in this study. However, the contribution of DC expansion and maturation to the improved efficacy was not directly examined. Future studies will focus on the proposed DC-associated mechanisms.

The combination of SOMV-9RE7 and alb-Flt3L enhanced antigen-specific CTL immune response in both peripheral blood and the spleen, which is essential for optimal systemic antitumor immunity. Specifically, increased circulating E7-specific CD8^+^ T cells indicate the dynamic mobilization of effector T cells for tumor infiltration. The expansion of total T cells and antigen-specific CTLs within the spleen, a major secondary lymphoid organ, suggests a highly coordinated central priming event. This enhanced clonal proliferation may provide a broader reservoir of tumor-targeting T cells and support durable efficacy.

MDSCs are known to expand in cancer and promote tumor progression through both immunosuppressive and non-immunological mechanisms [29]. Numerous studies have shown that MDSCs serve as crucial prognostic biomarkers for cancer progression and therapeutic effects [30]. In our study, circulating PMN-MDSCs and M-MDSCs were reduced only in the combination group, suggesting that the addition of alb-Flt3L is required to overcome systemic immunosuppression. These systemic changes are likely to work in a complementary way. The systemic expansion of antigen-specific T cells increases the effector pool, and the reduction in MDSCs may promote their function.

Memory formation may be another key factor contributing to therapeutic efficacy and durability. The expansion of CD8^+^ T_CM_ induced by the combination therapy suggests an enhanced memory reservoir. With high proliferative capacity and long-term persistence, this increased T_CM_ population may support continued effector T cell generation under persistent tumor antigen stimulation, which is relevant to the improved durability [31,32]. Although T_EM_ remained less abundant overall due to the late time point and high tumor burden, the increased frequency is still meaningful. This indicated a therapeutic potential even in a late-stage cancer. Overall, these findings suggest that SOMV-9RE7 and alb-Flt3L support long-term maintenance.

Ultimately, the enhanced antitumor immune responses were also reflected in the TME. The combination therapy significantly increased overall CD45^+^ TILs, indicating a more immune-infiltrated microenvironment. The increased proportion of CD8^+^ T cells in the combination group reveals a shift to a more cytotoxic immune pattern. In addition to quantitative expansion, this enhanced CTL response was also reshaped to a more tumor-specific phenotype. Together, these results suggest that the systemic immune alteration induced by the combination therapy is effectively translated into local antitumor immunity.

Although our study identified the combination of SOMV-9RE7 and alb-Flt3L as a promising strategy for durable and effective tumor control, some limitations still need to be acknowledged. Importantly, our findings primarily demonstrate the control of tumor growth and progression rather than the regression of established tumors. Additionally, efficacy in this study was evaluated exclusively in the TC-1 tumor model using the HPV E7 antigen. Further studies using larger established tumors, independent mouse models and distinct antigen targets will be needed to further evaluate efficacy and generalizability. In addition, the contributions of DC expansion and maturation will be investigated in this context to identify potential mechanisms.

In conclusion, our study reveals that combining vaccine SOMV-9RE7 with alb-Flt3L markedly improves antitumor efficacy and durability. This therapeutic benefit is associated with both systemic and local immune remodeling. Our work provides a strong foundation for the further development of therapeutic cancer vaccines.

## Data Availability

All relevant data are provided as Supplementary Information files.

## Supplementary Materials

The following supporting information can be downloaded at https://www.mdpi.com/article/10.3390/vaccines14080707/s1, Figure S1: Flow cytometry gating strategies for peripheral blood immune cell analysis; Figure S2: Flow cytometry gating strategies for splenic T cell analysis; Figure S3: Flow cytometry gating strategy for TME analysis.

## Abbreviations

The following abbreviations are used in this manuscript:

alb	albumin
CTL	cytotoxic T lymphocyte
DC	dendritic cell
Flt3L	Fms-like tyrosine kinase 3 ligand
HPV	human papillomavirus
MDSC	myeloid-derived suppressor cell
OMV	outer membrane vesicle
PBMC	peripheral blood mononuclear cell
PCR	polymerase chain reaction
T_CM_	central memory T cell
T_EM_	effector memory T cell
TME	tumor microenvironment
TIL	tumor-infiltrating lymphocyte

## References

[1] Pešut E., Đukić A., Lulić L., Skelin J., Šimić I., Milutin Gašperov N., Tomaić V., Sabol I., Grce M. (2021). Human Papillomaviruses-Associated Cancers: An Update of Current Knowledge. Viruses.

[2] Jensen J.E., Becker G.L., Jackson J.B., Rysavy M.B. (2024). Human Papillomavirus and Associated Cancers: A Review. Viruses.

[3] Burd E.M. (2003). Human Papillomavirus and Cervical Cancer. Clin. Microbiol. Rev..

[4] Aden D., Zaheer S., Khan S., Jairajpuri Z.S., Jetley S. (2024). Navigating the Landscape of HPV-Associated Cancers: From Epidemiology to Prevention. Pathol.—Res. Pract..

[5] Tomaić V. (2016). Functional Roles of E6 and E7 Oncoproteins in HPV-Induced Malignancies at Diverse Anatomical Sites. Cancers.

[6] Roman A., Munger K. (2013). The Papillomavirus E7 Proteins. Virology.

[7] Barnard P., McMillan N.A.J. (1999). The Human Papillomavirus E7 Oncoprotein Abrogates Signaling Mediated by Interferon-α. Virology.

[8] Li H., Zhan T., Li C., Liu M., Wang Q.K. (2009). Repression of MHC Class I Transcription by HPV16E7 through Interaction with a Putative RXRβ Motif and NF-κB Cytoplasmic Sequestration. Biochem. Biophys. Res. Commun..

[9] Shimu A.S., Wei H., Li Q., Zheng X., Li B. (2023). The New Progress in Cancer Immunotherapy. Clin. Exp. Med..

[10] Smalley Rumfield C., Roller N., Pellom S.T., Schlom J., Jochems C. (2020). Therapeutic Vaccines for HPV-Associated Malignancies. ImmunoTargets Ther..

[11] Zhao X., Zhang Y., Trejo-Cerro O., Kaplan E., Li Z., Albertsboer F., El Hammiri N., Mariz F.C., Banks L., Ottonello S. (2024). A Safe and Potentiated Multi-Type HPV L2-E7 Nanoparticle Vaccine with Combined Prophylactic and Therapeutic Activity. NPJ Vaccines.

[12] Martín E., Reina G., Carlos S. (2025). Advances in Therapeutic Vaccines Against HPV: A Review of Human Clinical Trials. Curr. Oncol..

[13] Zhou S., Gravekamp C., Bermudes D., Liu K. (2018). Tumour-Targeting Bacteria Engineered to Fight Cancer. Nat. Rev. Cancer.

[14] Kasinskas R.W., Forbes N.S. (2007). *Salmonella typhimurium* Lacking Ribose Chemoreceptors Localize in Tumor Quiescence and Induce Apoptosis. Cancer Res..

[15] Pawelek J.M., Low K.B., Bermudes D. (1997). Tumor-Targeted *Salmonella* as a Novel Anticancer Vector. Cancer Res..

[16] Schwechheimer C., Kuehn M.J. (2015). Outer-Membrane Vesicles from Gram-Negative Bacteria: Biogenesis and Functions. Nat. Rev. Microbiol..

[17] Chen S., Lei Q., Zou X., Ma D. (2023). The Role and Mechanisms of Gram-Negative Bacterial Outer Membrane Vesicles in Inflammatory Diseases. Front. Immunol..

[18] Kaparakis-Liaskos M., Ferrero R.L. (2015). Immune Modulation by Bacterial Outer Membrane Vesicles. Nat. Rev. Immunol..

[19] Cheng K., Zhao R., Li Y., Qi Y., Wang Y., Zhang Y., Qin H., Qin Y., Chen L., Li C. (2021). Bioengineered Bacteria-Derived Outer Membrane Vesicles as a Versatile Antigen Display Platform for Tumor Vaccination via Plug-and-Display Technology. Nat. Commun..

[20] Wang S., Cheng M., Chen C.-C., Chang C.-Y., Tsai Y.-C., Yang J.-M., Wu T., Huang C.-H., Hung C.-F. (2013). *Salmonella* Immunotherapy Engineered with Highly Efficient Tumor Antigen Coating Establishes Antigen-Specific CD8+ T Cell Immunity and Increases in Antitumor Efficacy with Type I Interferon Combination Therapy. Oncoimmunology.

[21] Wang S., Chen C.-C., Hu M.-H., Cheng M., Tu H.-F., Tsai Y.-C., Yang J.-M., Wu T.C., Huang C.-H., Hung C.-F. (2024). Arginine-Linked HPV-Associated E7 Displaying Bacteria-Derived Outer Membrane Vesicles as a Potent Antigen-Specific Cancer Vaccine. J. Transl. Med..

[22] Jin F., Xie L., Zhang H., Fan X., Tian J., Liu W., Xiao Y., Fan X. (2025). Dendritic Cells: Origin, Classification, Development, Biological Functions, and Therapeutic Potential. MedComm.

[23] McKenna H.J., Stocking K.L., Miller R.E., Brasel K., De Smedt T., Maraskovsky E., Maliszewski C.R., Lynch D.H., Smith J., Pulendran B. (2000). Mice Lacking Flt3 Ligand Have Deficient Hematopoiesis Affecting Hematopoietic Progenitor Cells, Dendritic Cells, and Natural Killer Cells. Blood.

[24] Wculek S.K., Amores-Iniesta J., Conde-Garrosa R., Khouili S.C., Melero I., Sancho D. (2019). Effective Cancer Immunotherapy by Natural Mouse Conventional Type-1 Dendritic Cells Bearing Dead Tumor Antigen. J. Immunother. Cancer.

[25] Wu S.Z., Lane R.S., Davidson C., Byrne A., Protti G., Marin I., Santosa E.K., Guanieri A., Kayser B.D., Huang H. (2025). Fibroblastic FLT3L Supports Lymph Node Dendritic Cells in the Interfollicular Niche. Nat. Immunol..

[26] Tu H.-F., Kung Y.-J., Lim L., Tao J., Hu M.-H., Cheng M., Xing D., Wu T.C., Hung C.-F. (2024). FLT3L-Induced Virtual Memory CD8 T Cells Engage the Immune System against Tumors. J. Biomed. Sci..

[27] Masopust D., Awasthi A., Bosselut R., Brooks D.G., Buggert M., Chamoto K., Cui W., Dong C., Farber D.L., Gebhardt T. (2026). Guidelines for T Cell Nomenclature. Nat. Rev. Immunol..

[28] Bhardwaj N., Friedlander P.A., Pavlick A.C., Ernstoff M.S., Gastman B.R., Hanks B.A., Curti B.D., Albertini M.R., Luke J.J., Blazquez A.B. (2020). Flt3 Ligand Augments Immune Responses to Anti-DEC-205-NY-ESO-1 Vaccine through Expansion of Dendritic Cell Subsets. Nat. Cancer.

[29] Dash S., Firmanty P., Chomczyk M., Mohanty V., Ma W., Baran N. (2026). Targeting MDSCs in Cancer: Emerging Immunotherapeutic and Metabolic Strategies. Front. Immunol..

[30] He S., Zheng L., Qi C. (2025). Myeloid-Derived Suppressor Cells (MDSCs) in the Tumor Microenvironment and Their Targeting in Cancer Therapy. Mol. Cancer.

[31] Wherry E.J., Teichgräber V., Becker T.C., Masopust D., Kaech S.M., Antia R., von Andrian U.H., Ahmed R. (2003). Lineage Relationship and Protective Immunity of Memory CD8 T Cell Subsets. Nat. Immunol..

[32] Klebanoff C.A., Gattinoni L., Restifo N.P. (2006). CD8+ T-Cell Memory in Tumor Immunology and Immunotherapy. Immunol. Rev..

