# Supplementary material for: An Outer Membrane Vesicle-Based Vaccine Combined with alb-Flt3L Promotes Durable Antitumor Immunity in HPV-Associated Cancer
Source: Vaccines (Basel). 2026 Aug 18;14(8):707. doi: 10.3390/vaccines14080707 (PMC13517856; doi:10.3390/vaccines14080707)
Supplement: Supplementary file 1 [file vaccines-14-00707-s001.zip › vaccines-4427601-supplementary.pdf]

Supplementary Materials

A.

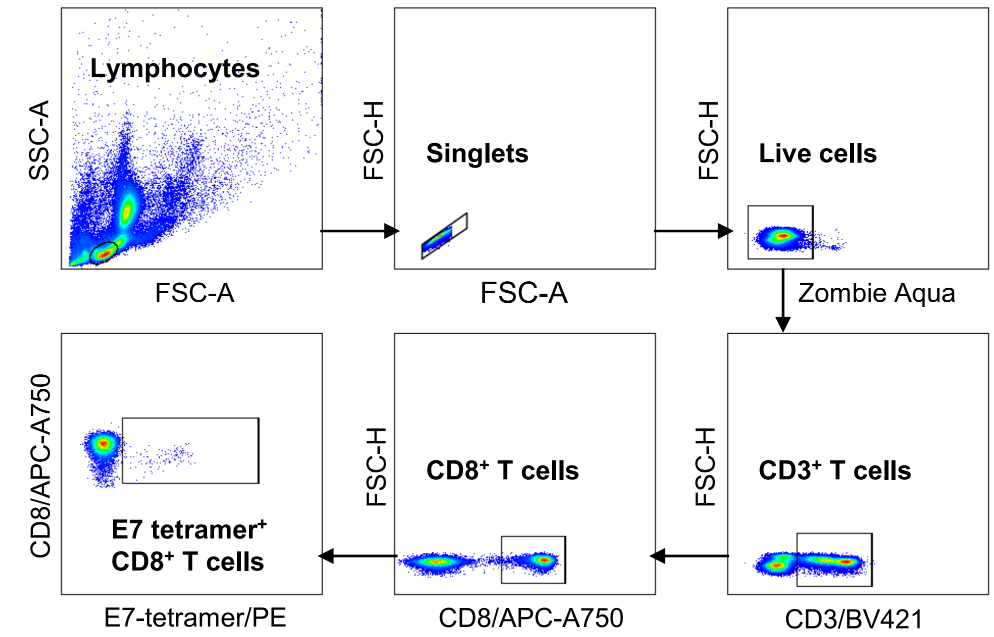

B.

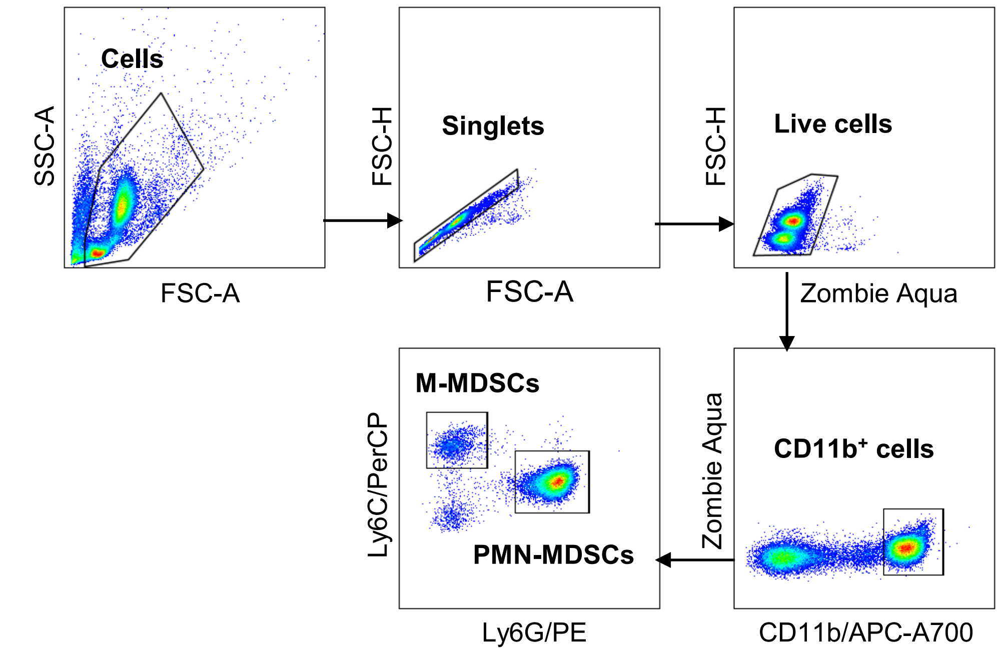

Figure S1. Flow cytometry gating strategies for peripheral blood immune cell analysis. (A) gating strategy for E7-specific CD8+ T cells. (B) gating strategy for MDSC populations. PMN-MDSCs and M-MDSCs were identified based on CD11b, Ly6C, and Ly6G expression.

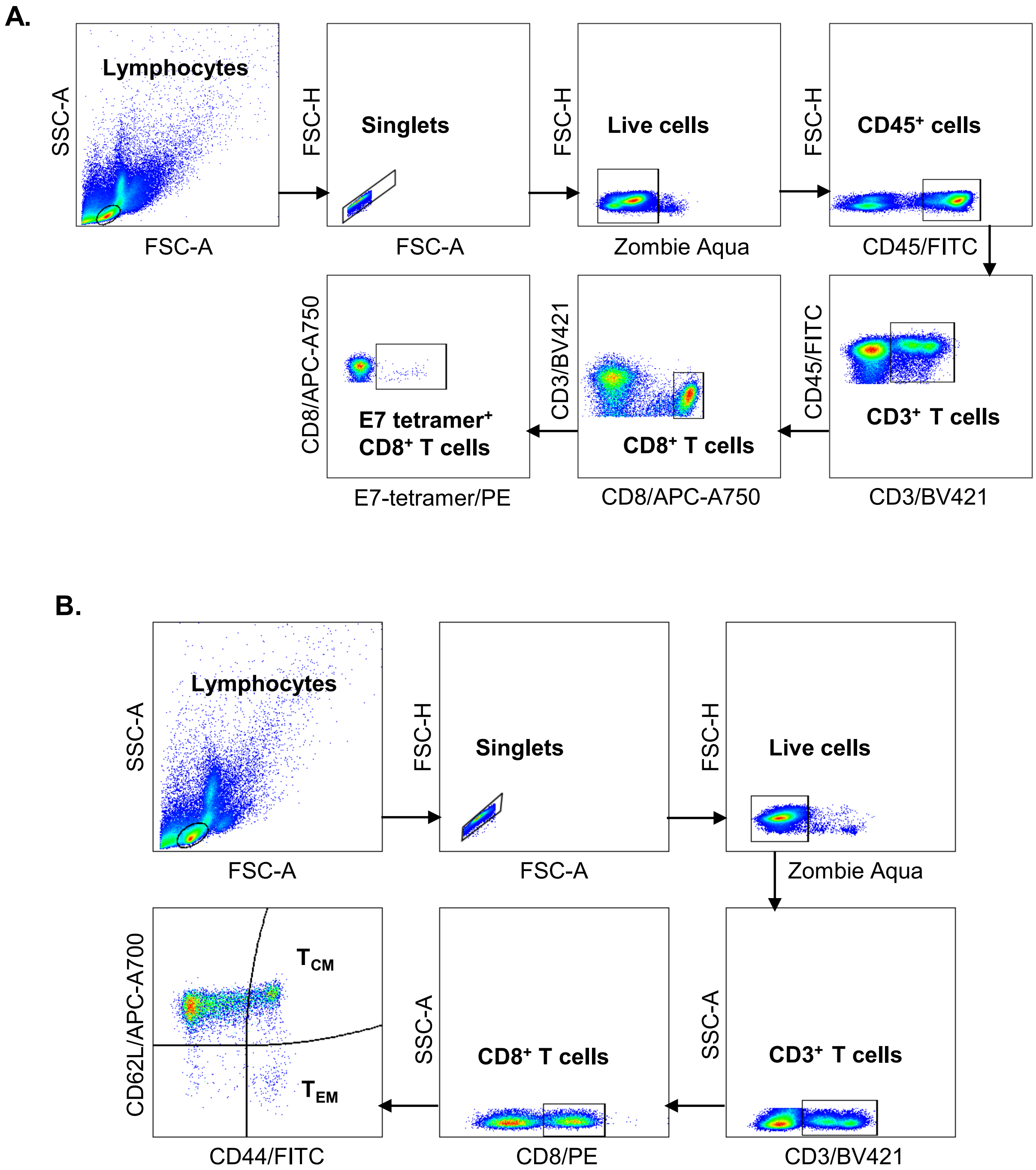

Figure S2. Flow cytometry gating strategies for splenic T cells analysis. (A) gating strategy for T cells and E7-specific CD8+ T cells. (B) gating strategy for CD8+ T cell memory subsets.

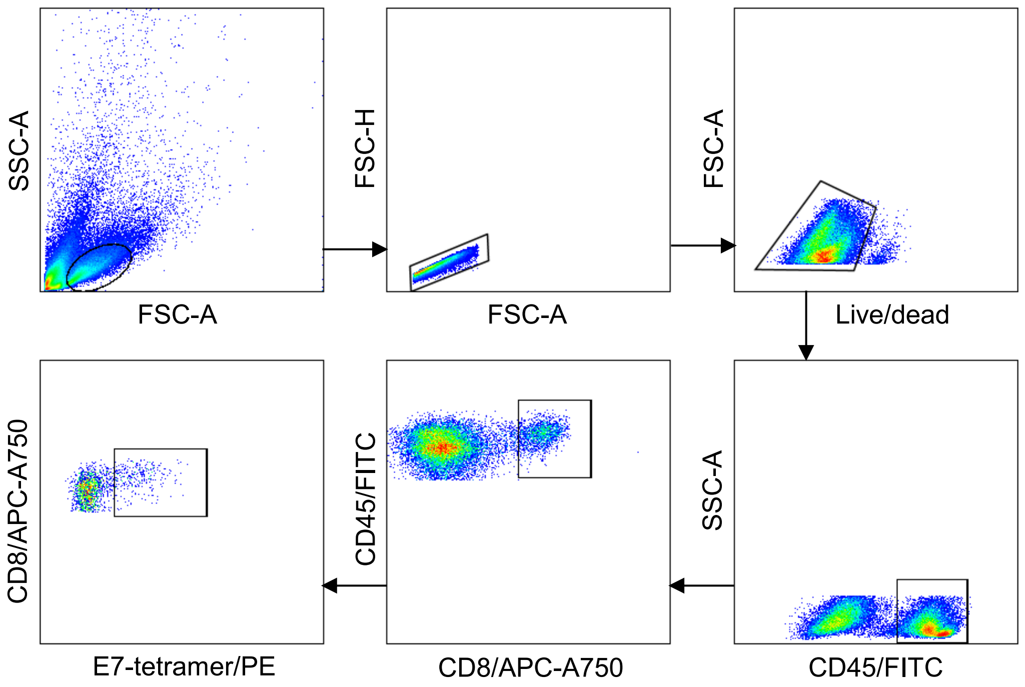

Figure S3. Flow cytometry gating strategy for TME analysis.
